# Focused, high accuracy 5-methylcytosine quantitation with base resolution by benchtop next-generation sequencing

**DOI:** 10.1186/1756-8935-6-33

**Published:** 2013-10-11

**Authors:** Dustin R Masser, Arthur S Berg, Willard M Freeman

**Affiliations:** 1 Department of Pharmacology, Pennsylvania State University College of Medicine, R130, 500 University Drive, Hershey, PA 17033, USA; 2 Department of Public Health Sciences, Pennsylvania State University College of Medicine, 500 University Drive, Hershey, PA 17033, USA; 3 Genome Sciences Facility, Pennsylvania State University College of Medicine, 500 University Drive, Hershey, PA 17033, USA

**Keywords:** 5-methylcytosine, Bisulfite sequencing, Next-generation sequencing, MiSeq

## Abstract

**Background:**

The growing interest in the role of epigenetic modifications in human health and disease has led to the development of next-generation sequencing methods for whole genome analysis of DNA methylation patterns. However, many projects require targeted methylation analysis of specific genes or genomic regions. We have developed an approach, termed BiSulfite Amplicon Sequencing (BSAS), for hypothesis driven and focused absolute DNA methylation analysis. This approach is applicable both to targeted DNA methylation studies as well as to confirmation of genome-wide studies.

**Results:**

BSAS uses PCR enrichment of targeted regions from bisulfite-converted DNA and transposome-mediated library construction for rapid generation of sequencing libraries from low (1 ng) sample input. Libraries are sequenced using the Illumina MiSeq benchtop sequencer. Generating high levels of sequencing depth (>1,000 ×) provides for quantitatively precise and accurate assessment of DNA methylation levels with base specificity. Dual indexing of sequencing libraries allows for simultaneous analysis of up to 96 samples. We demonstrate the superior quantitative accuracy of this approach as compared to existing Sanger sequencing methods.

**Conclusions:**

BSAS can be applied to any genomic region from any DNA source, including tissue and cell culture. Thus, BSAS provides a new validation approach for rapid and highly quantitative absolute CpG methylation analysis of any targeted genomic regions in a high throughput manner.

## Background

Epigenetic modifications, both histone modifications and DNA modifications (cytosine methylation and hydroxymethylation), are key regulators of the genomic structure and gene expression. Cytosine methylation in the CpG dinucleotide motif is essential for normal mammalian development [1] and control of imprinted genes [2]. The cytosines contained in CpG motifs are targeted for methylation on the 5-carbon site, often denoted as 5-methylcytosine, or 5-mC. CpG motifs are found throughout the genome, but at a lower frequency then would be expected by chance, and are often observed in clusters known as CpG islands [3]. Functionally, cytosine methylation is thought to regulate chromatin status and directly affect the ability of transcription factors to access DNA. Generally, in the context of gene promoters, hypomethylated CpGs are associated with active, constitutively expressed genes, while hypermethylated CpGs are associated with silenced genes [4].

A number of methods to assess and quantify DNA methylation at a genome-wide level, using either microarrays or sequencing, have been reviewed previously [5]. With the development of low-cost, high-output next-generation sequencing (NGS), 5-mC quantitation methods have predominantly transitioned to sequencing approaches. Many of the sequencing approaches utilize bisulfite conversion to differentiate methylated from unmethylated cystosines with base resolution [6,7]. Exposing DNA to sodium bisulfite deaminates unmethylated cytosines into uracil and these uracils are then replaced with thymines upon PCR amplification. Methylated cytosines, however, are protected from sulfonation and are not converted. Therefore, in sequence analysis of bisulfite-converted DNA, methylated cytosines appear as cytosine and unmethylated cytosines appear as thymines. Bisulfite DNA conversion and whole genome NGS provide wide coverage across the methylome. Whole methylome analyses remain complex, and resource intensive, which has also led to development of sequence enrichment strategies with antibodies or oligonucleotides (for example, MeDIP-seq, MethylCap), or digesting DNA with methylation sensitive restriction enzymes coupled to bisulfite conversion (reduced representation bisulfite sequencing (RRBS)) [5]. For highly targeted analyses (*that is*, selected genomic regions such as gene promoters, or validation of targeted regions from whole methylome analyses), traditional approaches of pyrosequencing or Sanger sequencing still predominate [8-11]. For hypothesis driven analyses, pyrosequencing and Sanger methods, while useful, are limited in their quantitative accuracy, read length and sample throughput. The quantitative deficiencies are due in part to the analog nature of their sequence outputs; light signal in pyrosequencing and fluorescent traces with Sanger sequencing and capillary electrophoresis [12].

Combining the benefits of bisulfite conversion, targeted amplification, tagmentation-based library construction and NGS we have developed a novel method, termed BiSulfite Amplicon Sequencing (BSAS), for targeted digital quantitation of DNA methylation. Utilizing BSAS, we validated the precision and accuracy of the method by targeted promoter region analysis in both rat and mouse whole genome methylation standards. Additionally, we analyzed methylation levels of known tissue differences in gene expression and promoter methylation and demonstrate the method’s ability to corroborate transcript expression levels with promoter methylation levels. These analyses were duplicated through bisulfite conversion and direct PCR Sanger sequencing to compare the performance of BSAS to an existing method of targeted methylation analysis. The application of BSAS is useful in hypothesis-driven epigenetic studies where regions of interest have been identified, and provides a rapid, accurate, and cost-effective method to quantify cytosine methylation with base specificity.

## Results

### BSAS method validation with rat and mouse methylation controls

**Figure 1:**
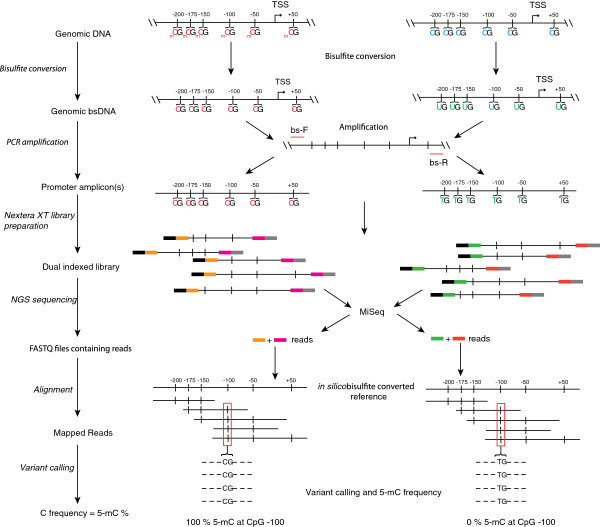
**Bisulfite amplicon sequencing (BSAS) method schematic.** Genomic DNA is bisulfite converted and subjected to bisulfite-specific PCR, using primers specific for bisulfite converted DNA (bs-F and R red lines). Amplicons are subjected to Nextera XT library preparation including dual indexing. Final libraries consist of a random insert of bisulfite converted, amplified DNA, capture probes (black and gray) and specific index sequences (orange, magenta, green, pink). These libraries are multiplexed and sequenced on the Illumina MiSeq. Demultiplexing separates the dual indexed reads from each sample (orange and magenta are one sample, green and pink represent the other sample). These reads are aligned to an *in silico* converted reference sequence, and variant calling is used to identify the percentage of 5-mC.

**Figure 2:**
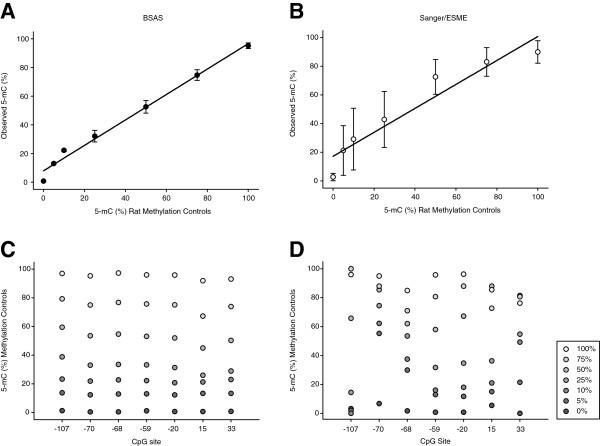
**Bisulfite amplicon sequencing (BSAS) and Sanger/epigenetic sequencing methylation (analysis (ESME) methylation quantitation of whole genome rat methylation controls. (A**,**B)** Standard curves were generated by BSAS and Sanger methylation quantitation and plotted out with expected percentage of methylation versus the actual quantified methylation. Points represent the mean of the seven CpG sites analyzed in the *Oprm1* promoter from each methylation standard (n = 3/control). Error bars = SD. **(C**,**D)** Average measured methylation for each standard plotted across each CpG site from BSAS and Sanger methylation quantitation (error bars not included for legibility).

**Figure 3:**
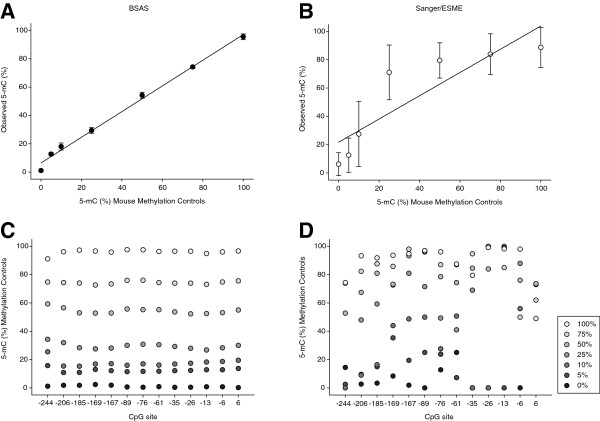
**Bisulfite amplicon sequencing (BSAS) and Sanger/epigenetic sequencing methylation analysis (ESME) methylation quantitation of whole genome mouse methylation controls. (A**,**B)** Standard curves were generated by BSAS and Sanger methylation quantitation and plotted out with expected percentage of methylation versus the actual quantified methylation. Points represent the mean of the 13 CpG sites analyzed in the *Rho* promoter from each methylation standard (n = 3/control). Error bars = SD. **(C**,**D)** Average measured methylation for each standard plotted across each CpG site from BSAS and Sanger methylation quantitation (error bars not included for legibility).

BSAS involves; (1) bisulfite conversion of genomic DNA to distinguish between methylated and unmethylated cytosines, (2) PCR amplification of regions of interest with PCR primers specific for converted sequences, (3) transposome mediated (Nextera XT) NGS library preparation technology, (4) sequencing the amplified regions on a benchtop sequencer (Illumina MiSeq), (5) alignment to *in silico* converted reference sequences, and 6) application of variant-calling algorithms for counting of cytosines and thymines at CpG sites for quantitative digital methylation analysis (Figure 1). Enzymatically generated whole genome rat and mouse methylation standards at mixed ratios (0, 5, 10, 25, 50, 75 and 100%) were analyzed for CpG methylation levels using BSAS (Figure 1) as well as the traditional Sanger sequencing approach with epigenetic sequencing methylation analysis (ESME). ESME was developed to eliminate the need for sequencing of multiple cloned amplicons per sample [13]. For the rat controls the mu opioid receptor, *Oprm1*, was used as the example region of interest and for the mouse standards the rhodopsin, *Rho* promoter region was analyzed. The MiSeq sequencing run for the rat standards produced a total of 4.21 million reads that passed the initial quality filter. Of these 1.96 million reads were mapped after overlapping read merging, quality trimming and elimination of PhiX control reads. For the mouse standards 10.81 million reads passed the quality filter, and 7.5 million reads were mapped after overlapping read merging, quality trimming and elimination of PhiX control reads. Bisulfite conversion efficiencies were confirmed to be >98% by examination of cytosine to thymine conversion for cytosines not in CpG motifs. Standard curves were generated from each set of methylation controls by taking the mean methylation level across the amplified region; 7 CpG sites from the rat controls, and 13 CpG sites from the mouse controls (Figures 2A and 3A). Both the rat and mouse standard curve BSAS data were able to fit linear lines of r^2^ = 0.99. Standard curves were also generated from each set of methylation controls analyzed with Sanger/ESME (Figures 2B and 3B). The Sanger/ESME control data were also able to fit linear lines with r^2^ = 0.90 and 0.73 for the rat and mouse controls, respectively. BSAS, for both sets of controls, was more accurate in methylation quantitation when compared to the standard curves generated from the Sanger/ESME method as evidenced from the higher correlation coefficients from the data generated from BSAS (r = 0.99) compared to the Sanger/ESME data (r = 0.88 and 0.96 mouse and rat, respectively). The precision of methylation quantitation was also greater in the data generated from BSAS when compared to the Sanger/ESME data. This is evident from the variation in methylation quantitation of the technical replicates (n = 3 per standard) of the generated standard curves for both sets of controls in both methods of methylation quantitation. The standard curves generated from the Sanger/ESME method have variations ranging from 5% to over 20% error in quantitation, while the BSAS method produced variation consistently less than 5% error.

The average measured methylation levels from both methods across technical replicates were then plotted out from each control across each CpG site for the mouse and rat methylation control sets. BSAS (Figures 2C and 3C) data was more accurate and less varied at each CpG site when compared to the Sanger/ESME method (Figures 2D and 3D). This was evident from the quantitation not overlapping at any of the controls at any CpG site for the BSAS data, while there is significant overlap of methylation quantitation across the CpG sites in the Sanger/ESME data. Additionally, the Sanger/ESME method was not sensitive enough to ensure all controls were represented at each CpG site. Because of this, there were missing values in the plots for the Sanger method.

By using whole genome methylation controls in two species to generate percentage of methylation standard curves, we demonstrated the ability of BSAS to consistently and accurately quantify CpG methylation when compared to traditional Sanger/ESME methylation quantitation. Under the assumption that the methylation standards were correct, mean squared error (MSE) statistical analysis for comparison of BSAS and Sanger/ESME demonstrated a 16-fold average increase in methylation quantitation accuracy, or decrease in error, across the mouse methylation controls. Quantitation of the rat controls yielded a fivefold average increase in accuracy (Table 1). BSAS demonstrated decreased error in methylation quantitation at each point in the standard curve from both species. Both methods were able to measure methylation from each control set, but BSAS was less varied and more sensitive in methylation quantitation, as shown by the ability to measure the percentage of methylation from each control set at each CpG site.

**Table 1:** Bisulfite amplicon sequencing (BSAS) accuracy improvement

**Control (% 5-mC)**	**Rat**	**Mouse**
0	5.41	30.51
5	2.61	1.53
10	1.79	10.01
25	3.05	1.03
50	12.17	25.47
75	7.33	40.79
100	3.81	4.78
Overall	5.16864	16.3067

Mean squared error (MSE) improvements of methylation quantitation from BSAS compared to Sanger/epigenetic sequencing methylation analysis (ESME) from each methylation control are presented. BSAS was more accurate at quantifying methylation at every control, and demonstrated an overall 16-fold decrease in error compared to Sanger/ESME across the mouse controls and 5-fold decrease in error across the rat controls.

### Rho promoter methylation tissue difference

**Figure 4:**
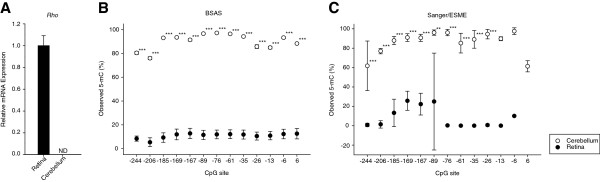
***Rho *promoter methylation in mouse retina and cerebellum. (A)** Quantitative PCR showed *Rho* mRNA expression to be high in the mouse retina (n = 4) while expression was not detected in the mouse cerebellum (n =4).** (B)***Rho* promoter methylation measured by bisulfite amplicon sequencing (BSAS) in both the cerebellum (white) and the retina (black) across 13 CpG sites in the *Rho* promoter. **(C)***Rho* promoter methylation measured by Sanger sequencing in the cerebellum and retina across 13 CpG sites in the *Rho* promoter. Error bars = SD. ***P <0.001, **P <0.002; t test.

We used mouse tissue to compare methylation quantitation in a biological scenario between BSAS and Sanger/ESME. We measured the *Rho* promoter methylation in retinal and cerebellar tissue, which contained 13 CpG sites (n = 4/tissue). Relative *Rho* mRNA expression, measured in the two tissues by quantitative PCR (qPCR), was found to be highly expressed in the retina and not detectable in the cerebellum (Figure 4A). Using the BSAS method, the sequencing run produced 8.22 million reads, and 6.54 million reads were mapped after read merging, quality trimming, and elimination of PhiX control reads. Bisulfite conversion efficiencies were confirmed to be >98%. The mean measured CpG methylation in the *Rho* promoter was found to be between 75% to 100% methylated at every CpG site evaluated in the mouse cerebellar tissue (Figure 4B). Conversely, the promoter CpG methylation in the *Rho* expressing retinal tissue was between 0% to 20%. Methylation levels were significantly higher (*P* <0.001) in the cerebellum than retina at every CpG site in the promoter. The same amplicons were sequenced using the Sanger/ESME (n = 4/tissue) (Figure 4C). The methylation levels measured in the cerebellar tissue were between 60% to 100% methylated, while the levels measured in the retina were between 0% to 30%. While BSAS produced statistically different methylation levels between the two tissues at each CpG site, the Sanger/ESME method did not. There were statistical significant differences at 10 of the 13 CpG sites measured (*P* <0.001 to 0.002), while the last 3 sites could not be tested due to the missing data caused by the lack of sensitivity from the Sanger/ESME method because of the analog quantitation and sequence coverage. In addition to every CpG site not being statistically significant, the variation in methylation quantitation, shown by standard deviation, was much greater in the Sanger/ESME data with standard deviation ranging from 5% to 30%. This error was much smaller in the BSAS methylation data, where there was at most 5% standard deviation.

### Sequence saturation and confidence interval

**Figure 5:**
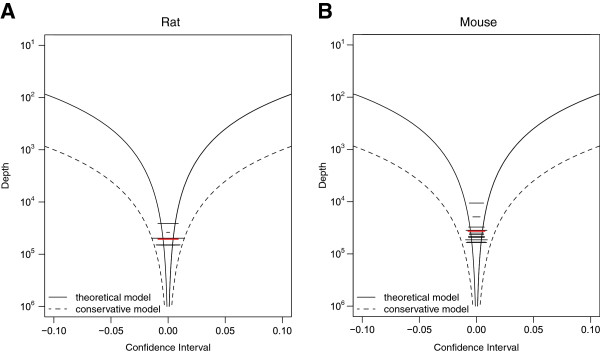
**Confidence interval sequencing saturation.** The theoretical confidence interval based on the binomial model of methylation quantitation for measurement of 50% cytosine methylation for both the **(A)** mouse and **(B)** rat methylation controls (solid black curves), where the empirical data are represented as horizontal lines (black) and the average of the empirical data (red line). A conservative confidence interval (dashed curves) is also shown, representing the theoretical model with 0.1*n* with *n* being the actual depth of the experimental data.

Overall, validation of BSAS was performed at high sequencing read depths (Additional file 1: Figure S2). To determine the optimal depth of sequencing required for quantitative accuracy, a binomial theoretical model was used to measure the confidence interval radius of methylation quantitation (Additional file 2: Figure S3). Empirical data visualized with the same model agreed with the theoretical model, with the average confidence interval radius difference being 0.00095. In order to assess the effect of sequencing depth on quantitation accuracy, we investigated the confidence interval for measuring 50% 5-mC, which based on the binomial theoretical model, has the largest confidence interval radius, at varying sequencing depths (10 to 1,000,000 ×) (Figure 5). The theoretical confidence interval shrinks with greater sequencing depth, as shown for both the rat (Figure 5A) and the mouse (Figure 5B) methylation controls. Additionally, the empirical mouse and rat data are represented as horizontal lines in their respective plots, at average sequencing depths of 10,000 to 100,000 ×. Based on the confidence interval, methylation quantitation is not greatly improved at sequencing read depths greater than 1,000 ×. Therefore, a sequencing depth of ≥1,000 × is sufficient for accurate methylation quantitation, while quantitation at lower sequencing depths might yield less accurate and wider spread data. Empirically, the rat and mouse data averages are slightly larger than the expected confidence interval for 50% methylation quantitation. Using a more conservative approach for calculating the confidence interval, the empirical data is well within the confidence interval radius for both the rat and mouse controls.

## Discussion

BSAS was developed to quantitatively and accurately measure 5-mC levels in genomic regions of interest. For many hypothesis-driven investigations or validation of genome-wide methylation studies, analysis of specific gene promoters, CpG islands or differentially methylated regions is needed. Combining existing approaches in bisulfite conversion with tagementation-based library preparation and benchtop sequencing produces a fast, accurate and customizable approach applicable to a wide variety of epigenetic studies. Using BSAS for targeted quantitative DNA methylation analysis, we demonstrated a 16-fold (mouse controls) and 5-fold (rat controls) decrease in the error of methylation quantitation over traditional direct bisulfite PCR amplicon Sanger sequencing and ESME. Whole genome DNA methylation standards were used to show this increase in accuracy of BSAS methylation quantitation across the dynamic range (from 0% to 100%) over the traditional approach in two regions from rat and mouse genomes. The increased accuracy in the BSAS method was attributed to the decrease in quantitation error over the traditional approach, seen in the standard curves generated. This decrease in error was most likely due to the digital quantitation of NGS as opposed to analog nature of the Sanger sequencing and the sequencing depth achieved in the BSAS method. *Rho* promoter methylation differences corroborated mRNA expression from retina and cerebellar tissue, with BSAS generating more precise data. In general, sequencing depth in the BSAS method ranged from 1,000 × saturation of the targeted region to well over 500,000 × saturation, at any given CpG site measured. These levels of depth were able to generate increased accuracy in quantitation of the methylation controls, in both the rat and mouse sets. Achieving 1,000 × sequencing depth was sufficient for accurate methylation quantitation. Using the theoretical model presented, the confidence interval does not improve significantly at depths greater than 1,000 ×. Therefore, when designing BSAS experiments, a target of 1,000 × would be a sufficient depth for accurate methylation quantitation. Our empirical data fit well within the theoretical model; however, there was a slight inflation of the confidence interval outside the expected for both the rat and mouse data sets. The source of error was most likely the methylation standards. Overall, the digital quantitation of BSAS improved the quantitation and statistical power over the Sanger method, which has an analog output with CpG methylation quantitation being a function of the area under the curves for the C and T traces. Thus, because BSAS is digital and reaches sequencing depths required for accurate quantitation, BSAS was superior to the Sanger method.

In addition to the better quantitation with BSAS, there were multiple benefits of using this method. There was no need to use sequencing primers as there is in pyrosequencing and direct bisulfite amplicon Sanger sequencing, which can limit these methods of sequencing in the quality of sequence obtained. The use of sequencing primers also limits these methods to looking at only one direction of one target region, limiting the throughput capabilities of pyrosequencing and Sanger sequencing reactions, and read length. Pyrosequencing is often limited to shorter reads (approximately 100 bp) requiring multiple tiled reads to achieve the coverage observed with BSAS [14]. In BSAS, the sequencing step is random and unbiased, thus multiple target genomic regions in one sample, and multiple samples, can be sequenced together on one flow cell. The use of cloning was not needed in BSAS; this reduced the overall time of the method considerably, as well as significantly improving the ease of library construction. Additionally, because of the digital nature of BSAS, when quantifying regions of interest, there is no need to generate a standard curve for each target. In the analog methods, standard curves are necessary for quantitation, because the quantitation is based on an assay signal, or output, not counting. This greatly reduces the amount of samples that need to be run with BSAS compared to pyrosequencing or Sanger.

Previously, targeted methylation analysis approaches, which have incorporated NGS into their protocols, depend on targeting of CpG sites by using hybridization arrays, padlock probe sets, and capture probes for whole genome methylation analysis [15-17]. Massively parallel PCR amplification with the Raindance technology followed by NGS has also been described [18]. These methods are well suited to ‘medium’ scale discovery but the complexity of the methods, requirement for additional specialized equipment and costs limit their application, especially for highly targeted studies with large numbers of samples. The BSAS approach only required standard molecular biology equipment and access to a benchtop sequencer. Where all of these methods excel is in their use of NGS for digital methylation quantitation. There are multiple reports profiling methylomes using NGS on tissues such as the human placenta [19], cancer cells [20], rodent animal models [21], rodent animal models [22], and disease states such as diabetes [22]. These findings break new ground in understanding DNA methylation across large regions of the genome, but a targeted approach of methylation analysis, like BSAS, is highly applicable to quantitative analysis of certain genes or regions, especially when a large number of samples are required. In particular, the rapid and cost effective nature of tagmentation-based library preparation and benchtop sequencing make BSAS an easily adopted method. An alternative approach to NGS-based focused methylation analysis is the MassARRAY mass spectrometry approach [23]. This approach has been used successfully for a number of studies but does require specific instrumentation, and non-sequence data can lead to ambiguity in determining base-specific methylation [24]. Therefore, whole methylome studies are useful for broad discovery efforts, while BSAS is a tool for answering hypothesis driven research questions of specific target genes or genomic regions identified in initial methylome analyses.

The BSAS method demonstrated the utility of the Nextera XT NGS library generation technology, which greatly reduces the amount of input DNA (1 ng), decreases library generation time (approximately 2 to 4 h), and increases the throughput of library generation by performing the protocol in a 96-well plate. Tagmentation also removes the need for stepwise DNA shearing, end repair, 3’ adenylation, and adapter ligation, combining these steps into one. The feasibility and benefits of tagmentation-based library preparations have been discussed previously [25]. Another benefit of NGS and Nextera XT library generation is the dual indexing libraries. Dual indexing allowed for a high level of sample multiplexing; 96 samples are capable of being multiplexed onto 1 single-lane flow cell. Within each sample, multiple target regions can be analyzed. The high level of multiplexing possible with BSAS, both in number of samples and regions of interest, increases the throughput over traditional methods. The current cost of tagmentation-based library construction is lower compared to ligation-based library construction methods. Provided sufficient flow-cell capacity, with BSAS additional time and costs are limited with increasing sample size. By comparison, Sanger and pyrosequencing require significant time and cost for each additional sample.

Supplemental to the simpler and more rapid library generation, we established the utility of the Illumina MiSeq in targeted DNA methylation analysis. While previous reports have used the MiSeq for high output cytosine modification validation [26] or comparing with traditional NGS library construction methods [25] we show the MiSeq as a tool for precise orthogonal absolute 5-mC quantitation and validation. The increased availability, short run times, and low cost of running benchtop sequencers make them an attractive tool for targeted analyses. The Illumina Miseq is currently capable of generating up to 15 Gb of sequence and 50 million paired-end reads passing quality filters on a single-lane flow cell. Additionally, sensitive optics and more precise base calling allow for low-diversity samples to be sequenced on the MiSeq. This decreases the amount of control libraries, like PhiX, that have to be added to improve base calling diversity, and reduces the amount of sequence lost to control sequences. Based on the performance of the method and the most conservative error rate model, more than 2 kb of target region(s) in 96 samples can be analyzed with high accuracy with 1 flow cell in a week by this method. Our findings show the utility of the MiSeq in future targeted epigenetic studies. Not only can the MiSeq generate enough data to sufficiently and accurately quantify DNA methylation, it can also be scaled in a cost-effective manner, depending on the amount of sequence (for example, number of samples, targets, and size of targets) through the use of the different size single-lane flow cells. Additionally, the ability of the MiSeq base-calling algorithms to handle low diversity samples, make it an excellent tool for sequencing bisulfite-converted, cytosine deficient DNA.

Limitations of the BSAS method, as described here, could arise from (1) PCR bias in the original bisulfite specific PCR, (2) bias in the transposome-mediated DNA fragmentation and adapter ligation, (3) and the inability of BSAS to distinguish between 5-mC and 5-hmC. PCR bias is not an issue when primers are generated properly. Parameters for designing bisulfite PCR primers that avoid bias have been addressed in the literature [27]. In our method, we observed no PCR bias in the data presented. Upon quantitative methylation analysis of the CpG sites targeted here, there was also no bias in the strand direction of the called variants. Additionally, the counts at the CpG sites were required to be in both the forward and reverse reads. Furthermore, we observed no GC bias in the tagmentation reaction within our amplicons sequenced using the Nextera XT protocol. As a result of targeting genomic regions, GC bias is less likely to occur. Previous studies validating the tagmentation-based library generation protocol demonstrated no GC bias in transposome tagmentation [28]. Additionally, previous studies have shown no differential GC bias between traditional ligation chemistry-based library generation methods when compared to tagmentation-based methods with bisulfite conversion [25]. The limitation of this method is the drop off in sequence depth at the end of the amplicons sequenced. This was attributed to the reduced likelihood of the transposome to insert at the ends of the amplicons. Finally, BSAS does not allow the quantitation of both 5-mC and 5-hmC. Our analysis may include both of these modifications. Future modifications of the protocol to be able to distinguish from these two modifications will be beneficial as studies of epigenetics progress. For example, the use of oxidizing agents, or glucosylation coupled to bisulfite conversion and analysis through the BSAS for separate 5-mC and 5-hmC quantitation [29,30].

## Conclusions

The BSAS method performed absolute 5-mC quantitation with high precision and accuracy, when compared to a traditional bisulfite sequencing method. Because of its targeted approach, highly quantitative data output, and high-throughput capabilities this method will be valuable in hypothesis driven epigenetic studies needing to investigate methylation changes in selected genomic regions where base resolution, high sample throughput and high quantitative accuracy is required.

## Methods

### DNA bisulfite conversion and bisulfite specific PCR

Nucleic acid was isolated from mouse retina and mouse cerebellum according to manufacturer’s protocol using Qiagen All-prep DNA/RNA coisolation (Qiagen, Germantown MD, USA). The Penn State animal facilities are fully accredited by the Association for Assessment and Accreditation of Laboratory Animal Care, and all animal procedures were approved by the Institutional Animal Care and Use Committee in compliance with the Public Health Service Policy on Humane Care and Use of Laboratory Animals and the National Research Council's Guide for the Care and Use of Laboratory Animals. DNA and RNA was quality checked by 260/280 absorbance ratio (E Nanovue spectrophotometer (GE Lifesciences, Uppsala, Swenden)). DNA was quantified using a fluorometric nucleic acid dye (Picogreen, Invitrogen/Life Technologies, Eugene, OR, USA) according to the manufacturer’s protocol and measured on a Spectromax M2 plate reader (Molecular Devices, Sunnyvale CA, USA). Whole genome enzymatically CpG dinucleotide methylated rat and mouse methylation controls were obtained from EpigenDx at mixed ratios (0% and 100%) of 0%, 5%, 10%, 25%, 75% and 100% methylation at 50 ng/μl (EpigenDx, Hopkinton, MA, USA). Controls methylation percentages are confirmed using pyrosequencing on gene-specific regions and global methylation assays. In all, 200 ng of methylated controls or 1 μg of genomic DNA (rat or mouse) were bisulfite converted using EZ DNA Methylation according to manufacturer’s protocol (Zymo Research, Irvine, CA, USA). Briefly, DNA was bisulfite converted for 14 to 16 h at 50°C (12 to 16 h of bisulfite conversion had no effect on bisulfite conversion efficiency, data not shown) and subsequently desulfonated, washed, and eluted in 10 μl elution buffer.

To amplify the *Oprm1* promoter region (-145 to +88 relative to transcriptional start site (TSS)) from the rat genome and *Rho* promoter region (-283 to +37 relative to TSS) from the mouse genome from bisulfite converted DNA the following primer sequences were used; rat Oprm1-F 5’-TTTTGGTTTTATTAGGGTTG-3’, rat Oprm1-R 5’-ACCAAAAACCAAATACTAAA-3’ [31], mouse Rho-F 5’-GAGATATTTTTTTTTTTTTTTATTTAAGGG-3’, mouse Rho-R 5’-AAAACACATAAAAATTAAAACCCTCTA-3’ [32]. PCR amplification of these regions was achieved by using Zymo*Taq* DNA polymerase, a DNA polymerase capable of amplifying low diversity DNA, specifically bisulfite converted DNA (Zymo Research, Irvine, CA, USA). Reactions were performed per manufacturer’s suggestions in 50 μl total volumes; 25 μl 2 × reaction buffer, 0.5 μl dNTP mix, 5 μl 10 μM forward and reverse rat Oprm1 primers, 2.5 μl 10 μM forward and reverse mouse Rho primers, 1 to 2 μl bisulfite template DNA, 0.4 μl Zymo*Taq* DNA polymerase, and ddH_2_O to 50 μl. Reaction conditions were: (1) Initial denaturation at 95°C for 10 minutes, (2) denaturation at 95°C for 30 s, (3) annealing at 44°C (rat Oprm1) or 50.1°C (mouse Rho) for 30 s, (4) extension at 72°C for 30 s, 35 cycles (steps 2 to 4), (5) final extension at 72°C for 7 minutes, and (6) and 4°C hold using a Mastercycler thermal cycler (Eppendorf, Hauppauge, NY, USA).

PCR products were purified by QIAquick PCR columns, to eliminate primers and enzymes, and eluted in 30 μl elution buffer (Qiagen, Germantown, MD, USA). PCR products were quantified using Picogreen (Invitrogen/Life Technologies, Eugene, OR, USA) fluorometric nucleic acid quantitation and measured on a Spectromax M2 plate reader (Molecular Devices, Sunnyvale CA, USA). PCR product sizes were confirmed through polyacrylamide gel electrophoresis on 6% Novex PAGE gels (Invitrogen/Life Technologies, Carlsbad, CA, USA). To visualize products, gels were soaked in ethidium bromide (0.5 μg/ml in ddH_2_O) and imaged on a Typhoon 9410 fluorescent scanner (GE Biosciences, Uppsala Sweden) at 532 nm excitation and 610 BP 30 emission filter.

### NGS library preparation

Dual indexed libraries were generated using Nextera XT library preparation technology according to manufacturer’s protocol (Illumina, San Diego, CA, USA). Purified PCR products were diluted to 0.2 ng/μl, and a total of 1 ng was used for library generation in a 96-well plate format. Transposome-mediated simultaneous DNA fragmentation and adapter ligation, that is, tagmentation, was performed at 55°C for 5 minutes. After the tagmentation reaction, indexing specific PCR primers were added two per well for unique dual indexing of the libraries for multiplex sequencing. Limited cycle-number PCR was performed to amplify the libraries and incorporate the index sequences to the libraries using the following reaction conditions; (1) 72°C for 3 minutes, (2) 95°C for 30 s, (3) 95°C for 10 s, (4) 55°C for 30 s, (5) 72°C for 30 s, 11 cycles (steps 3 to 5), (6) 72°C for 5 minutes, and hold at 10°C. Amplified libraries were purified using 30 to 50 μl AMPure XP beads (Beckman-Coulter, Brea, CA, USA), and eluted off the beads in 52.5 μl resuspension buffer (provided with Nextera XT).

Double-stranded libraries were quality checked on a High Sensitivity DNA Agilent chip run on the Agilent 2100 Bioanalyzer (Agilent Technologies) for size and molarity determination. Based on these metrics, libraries were diluted to 650 pM to 2 nM in 10 mM Tris with 0.5% Tween. Equimolar libraries were pooled in equal volumes for denaturation and dilution in HT1 (Illumina, San Diego, CA, USA) buffer. Briefly, 10 μl of pooled NGS library was mixed with 10 μl 0.2 N NaOH for 5 minutes, then the library was diluted to 20 pM in HT1 buffer. PhiX control libraries (Illumina, San Diego, CA, USA) were used to increase diversity of base calling during sequencing. Then, 10 nM stock of PhiX library was denatured in 0.2 N NaOH, then diluted to 20 pM in HT1 buffer. At this time, diluted, multiplexed libraries were mixed with diluted PhiX at 4:1 volume ratios. A final dilution to 8 to 12 pM was performed with HT1 buffer for a final volume of 1 ml. A volume of 600 μl was loaded onto the reagent cartridge for sequencing.

### Sequencing on Illumina MiSeq

Denatured and diluted libraries were sequenced on the Illumina MiSeq benchtop sequencer with the sequencing-by-synthesis technology per manufacturer’s protocol. Runs were set for ‘Generate FASTQ only’ workflow in Illumina Experiment Manager (Illumina, San Diego, CA, USA). Then, 300-cycle MiSeq v.1 reagent cartridges (Illumina, San Diego, CA, USA) were used to sequence libraries with paired-end, dual-indexing 151 cycles per read (2 × 151). Sequencing run monitoring was achieved through BaseSpace beta (basespace.illumina.com) (Additional file 3: Table S1). Data was demultiplexed on the MiSeq instrument automatically, and zipped FASTQ files were generated per sample, per read. Data was accessed either in the run analysis folder locally on the instrument, or through BaseSpace beta.

### NGS data analysis and digital methylation quantitation

FASTQ files were imported into CLC Genomics Workbench 5.5.2 or 6.0.2 (CLC Bio, Cambridge, MA, USA) as paired-end data retaining quality scores (NCBI/Sanger or Illumina 1.8 pipeline or later). A data analysis workflow was generated in CLC Genomics Workbench to automate methylation data analysis (Additional file 4: Figure S1). This workflow includes the following processes; paired-end overlapping read merge, read trimming on quality score (only reads containing base calls ≥Q30) and removal of reads with ≥1 ambiguous nucleotide. Merged and trimmed reads were then aligned to corresponding reference sequences that were bisulfite-converted *in silico* to allow stringent read alignment parameters in read mapping (C nucleotides remained in CpG dinucleotides for reads containing low methylation percentages). Mapping parameters were set up to score mismatches, insertions, and deletions at the highest possible penalty at a cost of 3 for each, with a nucleotide match always being a cost of 1. These scored reads were then filtered based on the minimum fraction of the read length that mapped to the reference, at 1, and minimum fraction of identity between the read and the reference, set to 0.9. These mapping parameters allowed for the retention of high-quality reads containing the majority of differences at CpG dinucleotide locations only. The bisulfite conversion efficiency was determined by the ratio of cytosine bases to converted reference thymine positions. Read mappings were ran through probabilistic variant detection analysis for measurement of C frequency in CpG positions, which correspond to the percentage of cytosine methylation. Analysis of data was the same between the 5.5.2 and 6.0.2 versions of CLC Genomics Workbench, with the exception of the variant calling analysis. Because version 6.0.2 calls variance dependent on linkage, two adjacent CpG sites would be measured as the same level of methylation, or cytosine frequency. The 5.5.2 version variant caller identifies variants independent of linkage, an important aspect of calling cytosine frequency for quantitation of adjacent CpG sites.

### Sanger sequencing and analog methylation analysis

PCR amplicons were sequenced by the Sanger method. Regions of interest were amplified using the same primers used in the bisulfite PCR with chain-terminating fluorescent dideoxy nucleotides. These products were then sequenced with an ABI 3130XL Capillary sequencer (Applied Biosystems/Life Technologies, Foster City, CA). Chromatogram traces (.abi) were analyzed using ESME, an automated open-source Sanger methylation data analysis software package developed for the Human Epigenome Project [13]. This software only analyzes CpG dinucleotides and measures intensity by the area under the curve from both the thymine trace and the cytosine trace, calculating the percentage of cytosine methylation by the value of cytosine intensity divided by the total intensity. It was developed for analyzing methylation for direct bisulfite PCR sequencing (http://www.epigenome.org/index.php?page=download).

### qPCR

qPCR analysis was performed as described previously [33,34] using the 7900HT Sequence Detection System (Applied Biosystems/Life Technologies, Foster City, CA), 384-well optical plates, and Assay-On-Demand (Applied Biosystems/Life Technologies, Foster City, CA) gene specific primers and probes (Mm Rho: Mm01184405_m1). SDS 2.2.2 software and the 2^-∆∆Ct^ analysis method were used to quantitate relative amounts of product using β-actin as an endogenous control. β-Actin levels were determined to be unchanged in an absolute quantitation experiment (data not shown).

### Statistics

Statistical analysis was performed using the standard parametric t test (α = 0.05, two tailed). An approximate confidence interval for sequence saturation is derived using a binomial probability model and is given by $\mu \pm 2\sqrt{\frac{\mu \left(1-\mu \right)}{n}}$, where *μ* is a given standard methylation ratio, and *n* is the number of sequenced reads covering the region of interest. Mean squared error (MSE) accuracy assessment was performed on quantified methylation controls to compare between BSAS and Sanger/ESME. To perform the MSE analysis, the methylation controls were assumed to be correct. The MSE ratio of the Sanger/ESME to BSAS quantitation methods were calculated and reported.

For the BSAS method, triplicate runs allow for an empirical calculation of a confidence interval for saturation along an amplicon with variable depths of sequenced reads. The empirical calculations are compared to confidence intervals based on the binomial probability model; for a standard methylation ratio of 0.5, the binomial-based confidence interval reduces to 0.5 $\pm \frac{1}{\sqrt{n}}$. The confidence intervals for varying sequencing depths were calculated and plotted.

## Abbreviations

BSAS: Bisulfite amplicon sequencing; NGS: Next-generation sequencing; RRBS: Reduced representation bisulfite sequencing; ESME: Epigenetic sequencing methylation analysis; MSE: Mean squared error; PCR: Polymerase chain reaction; TSS: Transcriptional start site.

## Competing interests

The authors declare that they have no competing interests.

## Authors’ contributions

DRM and WMF designed the method. DRM performed data generation. DRM and WMF analyzed and interpreted all data. ASB generated statistical modeling and performed MSE analysis on data. DRM, WMF, and ASB contributed to composing and editing the manuscript. All authors read and approved the final manuscript.

## Supplementary Material

Additional file 1: Figure S2 Depth plots across mouse and rat amplicons for bisulfite amplicon sequencing (BSAS). **(A)** Average sequencing depth plots across mouse amplicon and **(B)** rat amplicon for each methylation standard. Relative location along amplicon in reference to transcription start site. Click here for file

Additional file 2: Figure S3 Theoretical and empirical binomial model for methylation quantitation confidence interval radii of mouse methylation standards. Average empirical confidence interval radius is 0.0046, within the theoretical confidence interval radius. Average difference between the theoretical and empirical confidence interval was small, 0.00095. This model is based off the equation $\mu \pm 2\sqrt{\frac{\mu \left(1-\mu \right)}{n}}$ where *μ* is the methylation percentage and n is the sequencing depth. Click here for file

Additional file 3: Table S1 MiSeq run summaries. Single lane flow cell cluster densities, the percentage clusters passing filter, total paired reads passing filter, and total percentage reads above Q30. Click here for file

Additional file 4: Figure S1 Methylation quantitation workflow schematic. FastQ files are imported for workflow input. Reads are then merged to eliminate overlapping pairs. Sequences are then quality trimmed and mapped to a reference sequence. Variant detection is used to identify CpG sites and the percentage of cytosine methylation. Click here for file
