# Supplementary material for: Focused, high accuracy 5-methylcytosine quantitation with base resolution by benchtop next-generation sequencing
Source: Epigenetics Chromatin. 2013 Oct 11;6:33. doi: 10.1186/1756-8935-6-33 (PMC3907040; doi:10.1186/1756-8935-6-33)
Supplement: Additional file 1: Figure S2 — Depth plots across mouse and rat amplicons for bisulfite amplicon sequencing (BSAS). (A) Average sequencing depth plots across mouse amplicon and (B) rat amplicon for each methylation standard. Relative location along amplicon in reference to transcription start site. [file 1756-8935-6-33-S1.pdf]

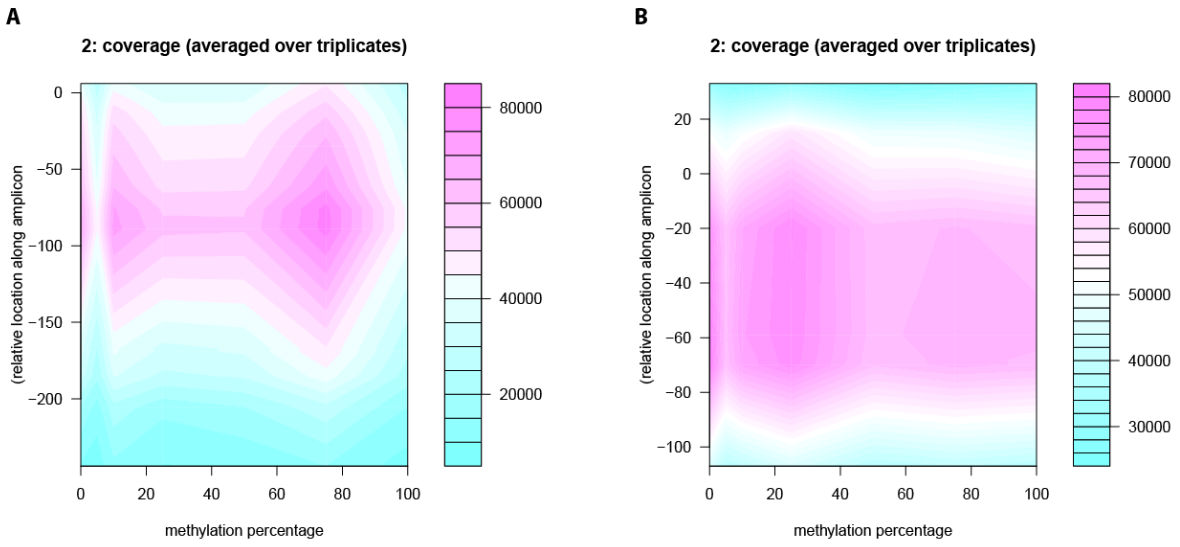

Supplemental Figure 2: Depth plots across mouse and rat amplicons for BSAS. A) Average sequencing depth plots across mouse amplicon and B) rat amplicon for each methylation standard. Relative location along amplicon in reference to transcription start site.
