# Supplementary material for: Focused, high accuracy 5-methylcytosine quantitation with base resolution by benchtop next-generation sequencing
Source: Epigenetics Chromatin. 2013 Oct 11;6:33. doi: 10.1186/1756-8935-6-33 (PMC3907040; doi:10.1186/1756-8935-6-33)
Supplement: Additional file 2: Figure S3 — Theoretical and empirical binomial model for methylation quantitation confidence interval radii of mouse methylation standards. Average empirical confidence interval radius is 0.0046, within the theoretical confidence interval radius. Average difference between the theoretical and empirical confidence interval was small, 0.00095. This model is based off the equation μ±2μ1−μn where μ is the methylation percentage and n is the sequencing depth. [file 1756-8935-6-33-S2.pdf]

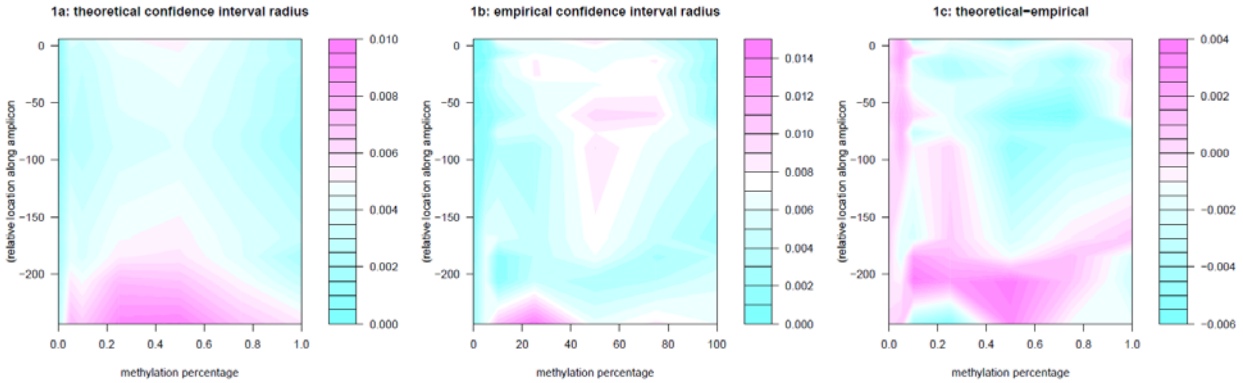

Supplemental Figure 3: Theoretical and empirical binomial model for methylation quantitation confidence interval radii of mouse methylation standards. Average empirical confidence interval radius is 0.0046, within the theoretical confidence interval radius. Average difference between the theoretical and empirical confidence interval was small, 0.00095. This model is based off the equation $\mu \pm 2\sqrt{\frac{\mu(1-\mu)}{n}}$ where μ is the methylation percentage and n is the sequencing depth.
