# Supplementary material for: Focused, high accuracy 5-methylcytosine quantitation with base resolution by benchtop next-generation sequencing
Source: Epigenetics Chromatin. 2013 Oct 11;6:33. doi: 10.1186/1756-8935-6-33 (PMC3907040; doi:10.1186/1756-8935-6-33)
Supplement: Additional file 3: Table S1 — MiSeq run summaries. Single lane flow cell cluster densities, the percentage clusters passing filter, total paired reads passing filter, and total percentage reads above Q30. [file 1756-8935-6-33-S3.pdf]

| **Run** | **Cluster Density ($K/mm^2$)** | **Clusters PF (%)** | **Total paired Reads PF (M)** | **Total % ≥Q30** |
|---|---|---|---|---|
| **Rat Standards** | 520 ± 4 | 96.83 ± 0.15 | 8.42 | 91.7 |
| **Mouse Standards** | 1287 ± 7 | 90.67 ± 0.89 | 21.62 | 82.4 |
| **RHO** | 929 ± 8 | 93.33 ± 0.75 | 16.44 | 83.1 |

**Supplemental Table 1:** MiSeq run summaries. Single lane flow cell cluster densities, the percent clusters passing filter, total paired reads passing filter, and total percent reads above Q30.
