# Supplementary material for: Focused, high accuracy 5-methylcytosine quantitation with base resolution by benchtop next-generation sequencing
Source: Epigenetics Chromatin. 2013 Oct 11;6:33. doi: 10.1186/1756-8935-6-33 (PMC3907040; doi:10.1186/1756-8935-6-33)
Supplement: Additional file 4: Figure S1 — Methylation quantitation workflow schematic. FastQ files are imported for workflow input. Reads are then merged to eliminate overlapping pairs. Sequences are then quality trimmed and mapped to a reference sequence. Variant detection is used to identify CpG sites and the percentage of cytosine methylation. [file 1756-8935-6-33-S4.pdf]

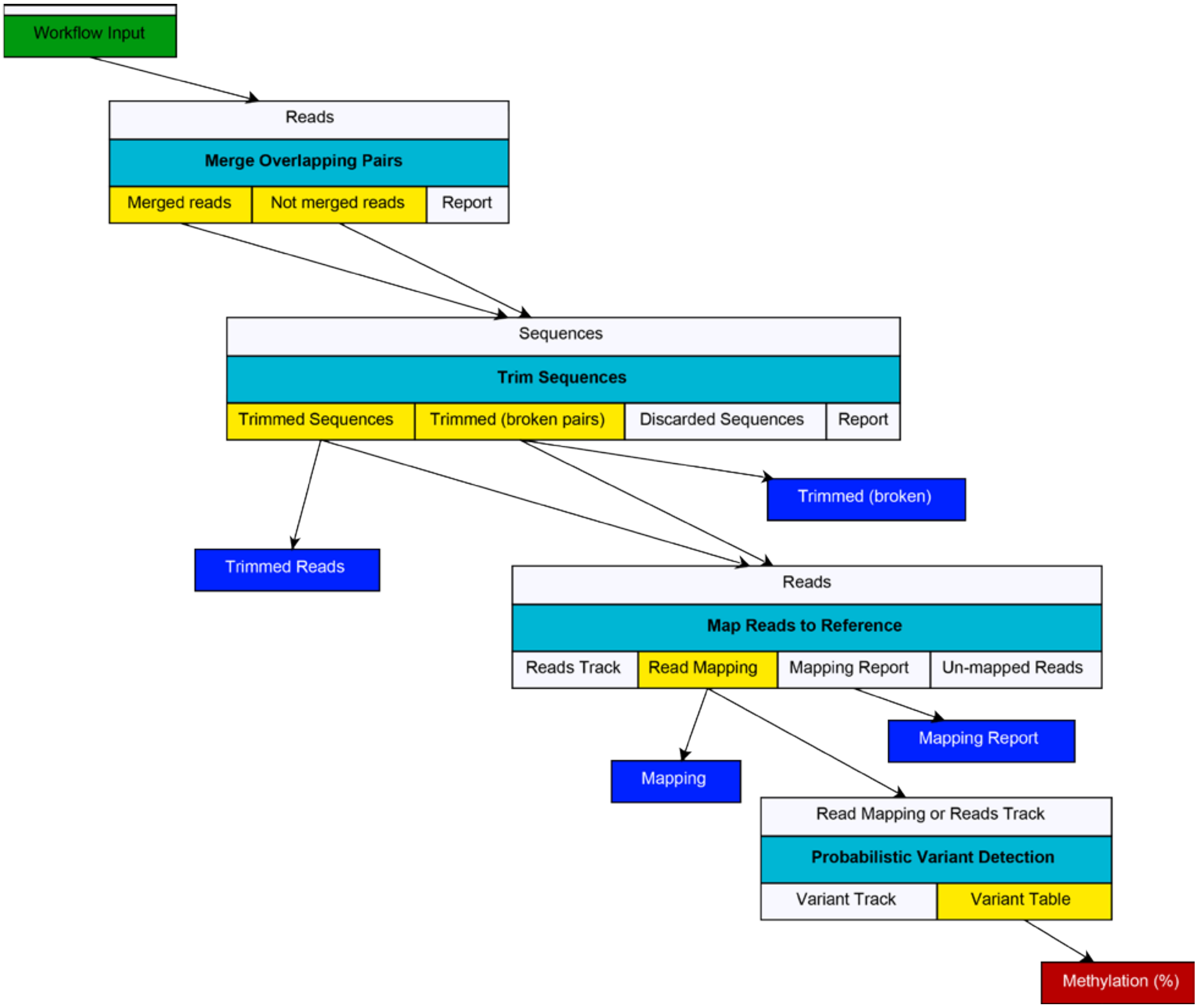

Supplemental Figure 1: Methylation quantitation workflow schematic. FastQ files are imported for workflow input. Reads are then merged to eliminate overlapping pairs. Sequences are then quality trimmed and mapped to a reference sequence. Variant detection is used to identify CpG sites and the percent cytosine methylation.
